# Agency, justice, and morality: exploring the intersection of belief in free will, the just world hypothesis, and health behavior

**DOI:** 10.3389/fpsyg.2026.1750010

**Published:** 2026-02-19

**Authors:** Gabriel Andrade

**Affiliations:** Ajman University, Ajman, United Arab Emirates

**Keywords:** agency, free will, health behavior, just-world hypothesis, moral judgment

## Abstract

This mini-review synthesizes empirical and theoretical insights on how belief in free will and the just world hypothesis jointly shape moral judgment and health behavior. It examines whether changes in these agency-related beliefs systematically affect prosociality, responsibility attribution, blame, and compassion, both independently and interactively. Experimental findings indicate that diminishing belief in free will increases dishonesty and reduces empathy, while stronger just world beliefs are linked to victim-blaming, harsher moral judgment, and rationalization of inequality. The review also considers how these beliefs extend to health, where strong agency convictions can promote motivation, self-regulation, and healthier lifestyles, yet simultaneously heighten stigma toward individuals facing obesity, mental illness, or other health challenges. Drawing on cross-cultural and health psychology frameworks, the paper argues that culturally sensitive interventions are needed to harness the motivational benefits of agency while reducing blame, stigma, and exclusion in health and moral policy.

## Introduction

Belief in free will—the perception that individuals possess the capacity to make autonomous choices—serves as a cornerstone for much of moral psychology and social cognition. Central to debates is whether suspending this belief alters behavior, responsibility, and judgment, with foundational research suggesting that belief in free will supports self-control, ethical conduct, and prosocial attitudes (Buttrick et al., 2020; Katzir and Genschow, 2022a; Schooler et al., 2014). Parallel to this is the belief in a just world, positing that outcomes in life reflect merit and virtue—those who do good receive good—a thesis extensively examined in classic work by Melvin Lerner and contemporary theorists (Hadarics and Kende, 2025). The philosophical logic connecting these beliefs is clear: if individuals are expected to “deserve” outcomes, the premise of free choice is necessary. However, empirical research on their behavioral concordance and potential interactive effects has only recently emerged.

Building on these foundations, the present mini-review is guided by several key research questions. First, it asks whether changes in belief in free will or belief in a just world lead to systematic shifts in moral and prosocial behavior, such as increased dishonesty or reduced compassion. Second, it explores whether these beliefs are psychologically interlinked or whether their effects are additive and independent, paying special attention to the circumstances in which undermining one may amplify or attenuate the effects of the other. Third, the review investigates whether and how modifications in these beliefs reshape broader forms of social judgment, including responsibility attribution, blame, victim-blaming, and empathy when confronting the suffering or failings of others. As a fourth guiding question, the review considers whether these philosophical and psychological constructs have implications for health-related behavior and attitudes—particularly, whether agency-related beliefs foster healthier lifestyles or, conversely, promote stigma and reduce support for those facing health challenges. Addressing these inquiries will help clarify the mechanisms by which free will and just world beliefs manifest in everyday conduct, as well as their implications for interventions designed to balance personal responsibility with compassion.

## Discussion

### The behavioral effects of belief in free will and belief in a just world

Empirical findings strongly suggest that priming disbelief in free will leads to measurable changes in ethical and social conduct. Pioneering studies showed that participants primed with deterministic perspectives (the notion that free will is illusory) engaged in significantly more cheating and dishonest behavior than controls by Vohs and Schooler (Shariff et al., 2008). Meta-analytic reviews and experimental replications (Caspar et al., 2017; Katzir and Genschow, 2022a) reinforce these claims, showing increases in antisocial tendencies (e.g., cheating, racial prejudice, aggression) when belief in free will is diminished. The larger psychological mechanism is thought to be lowered sense of personal control and diminished self-regulation—a notion supported by neuroscience research linking free will beliefs to neural correlates of intentional action and error processing (Bonicalzi and Haggard, 2019; Watson, 1987).

Further, Clark et al. (2014) demonstrated that belief in free will increases after reflecting on immoral actions by others, suggesting a dynamic interplay between one’s social environment and these foundational beliefs. Other research has found that weakening free will beliefs prompts a shift toward automatic, impulsive responses, reducing deliberate moral reasoning and increasing selfish tendencies in cooperative tasks (Burns and Bechara, 2007; Wertenbroch et al., 2008).

Belief in free will also affects how individuals judge others. Experimental studies reveal that belief in free will intensifies the correspondence bias—the tendency to attribute behavior to dispositional causes rather than situational ones—resulting in harsher punishment and greater certainty when making moral judgments about others (Katzir and Genschow, 2022b). Conversely, reducing belief in free will leads to less judgmental attitudes and more situational attributions for other people’s actions (Everett et al., 2021; Hannikainen et al., 2019). This has strong implications for both criminal justice and everyday moral reasoning.

The belief in a just world (BJW) similarly exhibits robust behavioral associations. The theory of the just world hypothesis was first introduced by social psychologist Melvin Lerner in the 1960s, who observed that people have a strong tendency to believe that the world is fundamentally fair and that individuals get what they deserve. Lerner’s seminal experiments demonstrated that when people witnessed an innocent person suffering, such as in the case of a confederate being (apparently) shocked in a laboratory setting, observers would begin to derogate or blame the victim rather than accept the randomness of injustice; this was especially pronounced when observers were powerless to assist (Lerner and Miller, 1978). These findings, detailed further in Lerner (1980) influential book, *The Belief in a Just World: A Fundamental Delusion*, established BJW as a pervasive cognitive bias that provides psychological comfort by imposing moral order on a chaotic reality, but also leads to problematic social outcomes such as victim-blaming and rationalization of inequality. Subsequent studies have consistently shown that stronger just world beliefs predict greater victim-blaming, harsher moral judgments, and resistance to recognizing the structural causes of misfortune (Montada and Lerner, 1998).

General BJW correlates with greater harshness in social attitudes, higher propensity for antisocial behavior (Wenzel et al., 2017), and increased tendency to judge others as deserving their fate (Kong et al., 2021). On one hand, this belief may exert an adaptive influence, aiding individuals in meaning-making, resilience, and positive adjustment during adverse circumstances (Wu et al., 2011). On the other, it may foster less compassion and greater victim-blaming, especially when interpreting other people’s misfortunes or illnesses. Some research has found that individuals with high BJW maintain self-esteem even after reminders of personal unfairness (Bègue, 2005; Tatsi and Panagiotopoulou, 2023), suggesting a self-protective function but also a diminished sense of guilt about one’s conduct.

When individuals hold a strong belief in a just world, they may rationalize any negative actions they commit against others by assuming those individuals must ultimately deserve their fate, reducing feelings of guilt or responsibility for causing harm. This cognitive bias not only shields the believer from moral discomfort, but it also leads to greater justification of antisocial or dishonest behaviors, as one may conclude that personal actions do not truly inflict injustice—since justice, in their worldview, always prevails independently.

In health contexts, the combined influence of free will and just world beliefs is especially evident in everyday reactions to lifestyle-related conditions such as type 2 diabetes, cardiovascular disease, or substance use disorders. A clinician who strongly endorses free will may interpret non-adherence to diet or medication as a straightforward failure of effort, concluding that the patient simply “chose” not to comply, rather than probing for barriers such as low health literacy, chaotic work schedules, or unaffordable prescriptions. By illustrating how abstract agency-related beliefs shape concrete clinical judgments, this example helps bridge the move from general moral evaluation to the later theoretical analysis of how these beliefs support or undermine compassion, thereby preparing the ground for questions about their broader psychological functions and potential interaction.

Similar dynamics appear in public responses to emerging health threats or chronic conditions that are partially behavior-related but also strongly shaped by social determinants. During outbreaks of infectious disease, for instance, people who hold strong free will beliefs may focus on individual compliance with hygiene or vaccination recommendations, framing infection as the result of negligent personal decision-making (Claudy et al., 2022). Making explicit how just world assumptions can then encourage the view that those who become ill “must have been careless” shows how agency beliefs extend from interpersonal blame to policy-relevant attitudes about vulnerability and responsibility, thereby motivating the ensuing discussion about when such beliefs foster harshness versus when they might be reconfigured to support more equitable, context-sensitive judgments.

Free will and just world beliefs can also shape attitudes toward access to healthcare and the distribution of limited resources. In debates over eligibility for organ transplantation, intensive care, or costly new treatments, those who strongly endorse personal agency may argue that patients whose illnesses are perceived as “self-inflicted” (for example, cirrhosis in heavy drinkers or lung disease in long-term smokers) should be deprioritized because they are more responsible for their condition (Ubel et al., 2001). Explicitly showing how this distributive logic draws on both desert-based justifications and assumptions about controllability underscores why the subsequent theoretical section must examine not only whether these beliefs co-occur, but how their joint operation structures judgments about desert, blame, and aid across domains such as health, justice, and social policy.

### Theoretical foundations and areas for future research

Philosophically, if one asserts that people “get what they deserve,” then it presupposes agency and freedom in their actions—a foundational connection between free will and just world belief. An intriguing area for future empirical work is whether disrupting BJW has effects on immoral behaviors similar to those seen when suppressing free will beliefs; i.e., might people with a diminished sense of justice feel less need to adhere to moral norms? Additionally, further research is warranted to explore whether suppressing free will beliefs universally makes individuals less judgmental—not just in terms of their own moral standards, but in the evaluation of others’ actions. The bidirectional consequences on compassion, blame, and social responsibility remain ripe for new empirical study.

Research demonstrates a moderate positive correlation between belief in free will and belief in a just world (BJW), theorizing that both function to sustain a sense of order, predictability, and personal agency in life (Bargh, 2008; Carey and Paulhus, 2013; Caruso, 2019; Genschow and Vehlow, 2021). While the philosophical connection suggests that just outcomes presuppose that people can choose their actions, empirical work confirms that individuals who endorse one are more likely to endorse the other. This has led recent literature to regard belief in free will and BJW as part of a broader “agency-control complex” supporting self-regulation, coping, and meaning-making.

BJW also shapes attitudes toward others: it underpins the tendency to view victims as blameworthy and to perceive social inequality as deserved, contributing to reduced compassion and increased harshness toward those experiencing misfortune (Hafer and Sutton, 2016). Thus, while BJW can empower self-regulation and hopefulness, it can also undermine empathy and reinforce punitive attitudes.

Although much research has emphasized the risks of suppressing belief in free will—pointing to increased antisocial or impulsive behaviors—a complementary perspective is that disbelieving in free will could foster greater compassion and empathy toward others. When individuals view actions and life outcomes as being substantially shaped by factors beyond personal control, they may become less inclined to judge or blame others, attributing misfortune and moral failings to circumstance rather than character. This reduction in harsh, dispositional judgment could promote a more tolerant and understanding social climate, particularly toward those struggling with adversity, mental health challenges, or poverty. Further empirical research is warranted to test these hypotheses.

Despite its potential social benefits, the positive effects of free will disbelief remain underexplored in empirical work. Future studies could investigate whether decreasing belief in free will consistently leads to increases in compassion, forgiveness, and supportive behaviors across different domains. Such research would help clarify whether the attenuation of harsh judgment goes hand-in-hand with reduced punitive attitudes and more inclusive social norms, or if specific contexts and individual factors—such as empathy or moral sensitivity—are key moderators of these effects. Expanding this line of inquiry may offer valuable insights for interventions aimed at reducing stigma and fostering greater social cohesion.

The influence of free will and just world beliefs on compassion and judgment is likely contingent on context and may vary significantly across cultures and demographic groups. Cross-cultural research has found that beliefs in free will differ between individualistic societies (Kushnir, 2018; Lam and Yeung, 2025; Wente et al., 2016; Wisniewski et al., 2019), where autonomy and personal responsibility are strongly emphasized, and collectivist cultures, which tend to interpret actions as more socially embedded and influenced by external factors (Chernyak et al., 2019; Robertson, 2017). Just world beliefs also show such variation, shaped by cultural narratives about fairness, fate, and social structure (Furnham, 1985; Furnham and Procter, 1989; Pedersen and Strömwall, 2013). As a result, the degree to which these beliefs interact to produce more compassionate or less judgmental attitudes toward others may depend heavily on whether a culture prioritizes personal agency or collective outcomes.

Moreover, this variability suggests that interventions or policies aimed at increasing compassion or decreasing moral harshness by addressing free will or just world beliefs must be culturally sensitive. What fosters empathy and understanding in one cultural setting may reinforce blame or detachment in another. Exploring these demographic and contextual moderators is crucial for future research, as it will provide clearer insights into how belief systems translate into moral attitudes and behavior across diverse social environments.

### Health behaviors: belief in agency and responsibility

The influence of both free will and just world beliefs extends beyond moral judgments and social interactions. Based on these theoretical insights and empirical findings, it is essential to explore how these beliefs may affect health-related behaviors and perceptions. Individuals who strongly endorse free will generally report greater perceived behavioral control and responsibility, which can lead to healthier choices such as more physical activity and more dietary restraint (St Quinton and Crescioni, 2024a, 2024b). Similarly, just world beliefs can foster a sense of autonomy and self-regulation, encouraging individuals to adopt and maintain positive health habits in the belief that responsible behaviors will yield deserved benefits.

At the same time, both beliefs can also shape attitudes toward others’ health outcomes. Attribution of obesity and other health conditions to individual moral responsibility is fundamentally rooted in the belief in free will. It remains to be studied if those who strongly endorse free will are more likely to perceive adverse health outcomes, such as obesity or mental illness, as the result of personal choices and controllable actions, rather than as consequences of genetic, environmental, or social circumstances. This perception may increase the likelihood of stigmatizing and blaming individuals struggling with such issues, as it frames their health status as a reflection of their willpower or character.

Conversely, understanding how these beliefs shape healthcare attitudes and patient interactions is crucial for advancing compassionate practice. Interventions targeting reductions in stigma may need to address underlying free will assumptions that may amplify harsh judgment and inhibit empathy. Investigating these links will not only improve our understanding of how psychological agency impacts health-related behavior and wellbeing, but also inform policies to promote more constructive, supportive public attitudes and effective healthcare delivery.

The relationship between free will beliefs and health behavior is multifaceted, serving not only as a source of blame or stigma but also as a catalyst for motivation and self-care. Individuals who believe strongly in free will are more likely to perceive control over their health outcomes and thus show increased commitment to behaviors such as regular physical activity, healthy eating, and restraint in risky conduct. This heightened sense of agency encourages persistence and personal responsibility, shaping health behaviors in ways that can foster overall well-being. The belief in a just world can further strengthen this effect by boosting the conviction that responsible choices will yield deserved benefits, supporting resilience and goal-directed action.

These motivational processes are grounded in the Theory of Planned Behavior (TPB), a prominent framework in health psychology (Ajzen, 2011; Conner et al., 2002; Godin and Kok, 1996). TPB posits that human behavior is shaped by three core components: attitudes toward the behavior, subjective norms (the influence of societal or peer expectations), and perceived behavioral control (an individual’s perception of their ability to perform the action). Belief in free will is closely tied to the perceived behavioral control aspect: the more a person believes they are capable of controlling their own actions and outcomes, the more likely they are to form strong intentions and carry out health-promoting behaviors. Conversely, deterministic or fatalistic worldviews undermine perceived behavioral control, resulting in less motivation to self-regulate and a higher likelihood of unhealthy habits. Thus, free will and just world beliefs intersect with TPB to help explain why some individuals are more proactive in caring for themselves—while simultaneously highlighting the potential risks of stigmatizing those who struggle with health issues.

People who strongly believe in free will may feel a deeper sense of agency over their bodies and life outcomes, leading them to adopt healthier routines, persist in the face of setbacks, and assume greater responsibility for maintaining their wellbeing. When combined with the belief in a just world, this mindset can reinforce the conviction that healthy actions will yield positive results and that individuals can “create” their own deserved outcomes.

This dual effect means that free will and just world beliefs can have both empowering and potentially problematic consequences: on one hand, promoting autonomy, motivation, and personal achievement; on the other, fostering judgment or stigma toward those perceived as failing to exercise their will. Understanding this dynamic helps clarify critical points for intervention, suggesting that health promotion efforts should leverage the motivational aspects of agency while strategically addressing the risk of increased blame and reduced compassion for those facing health challenges outside their control.

## Conclusion

The review highlights that belief in free will and belief in a just world are key psychological constructs shaping both moral conduct and health-related behavior. These beliefs are shown to support agency, self-control, and personal responsibility, which can motivate healthier choices and greater persistence in maintaining well-being. However, the same cognitive frameworks can foster stigma and judgment toward those perceived as failing to exercise adequate willpower or “deserving” their struggles, thus potentially reducing compassion and social support in health contexts.

This dual impact has important implications for policy in healthcare and public health. On one hand, harnessing the motivational potential of free will and just world beliefs may empower individuals to embrace preventive health behaviors and adhere to treatment regimens, reinforcing autonomy and resilience. On the other, policy makers, practitioners, and educators must remain vigilant against the risk of amplifying blame or moralizing health outcomes. Culturally sensitive interventions are crucial—policies and campaigns should foster responsibility and agency while explicitly countering stigma and promoting empathetic attitudes toward those facing medical or psychological challenges.

Ultimately, understanding how free will and just world beliefs function across diverse populations and settings is essential for crafting effective interventions. Future research can further clarify when these beliefs reinforce positive engagement, when they risk judgmentalism, and how nuanced educational and policy measures can maximize public well-being—sustaining motivation and fairness, yet reducing bias and exclusion. Such integration of psychological insight into health policy can help balance the ideals of personal agency with the need for community, compassion, and justice.
